# Development and Validation of a Prognostic Model for Predicting Overall Survival in Patients With Bladder Cancer: A SEER-Based Study

**DOI:** 10.3389/fonc.2021.692728

**Published:** 2021-06-17

**Authors:** Wei Wang, Jianchao Liu, Lihua Liu

**Affiliations:** ^1^ Institute of Military Hospital Management, The Chinese PLA General Hospital, Beijing, China; ^2^ Department of Rehabilitation Medicine, Qingdao Special Servicemen Recuperation Center of People’s Liberation Army (PLA) Navy, Qingdao, China

**Keywords:** prognosis, nomogram, bladder cancer, Surveillance Epidemiology and End Results database, risk factor

## Abstract

**Objective:**

To establish a prognostic model for Bladder cancer (BLCA) based on demographic information, the American Joint Commission on Cancer (AJCC) 7th staging system, and additional treatment using the surveillance, epidemiology, and end results (SEER) database.

**Methods:**

Cases with BLCA diagnosed from 2010–2015 were collected from the SEER database, while patient records with incomplete information on pre-specified variables were excluded. All eligible cases were included in the full analysis set, which was then split into training set and test set with a 1:1 ratio. Univariate and multivariate Cox regression analyses were conducted to identify prognostic factors for overall survival (OS) in BLCA patients. With selected independent prognosticators, a nomogram was mapped to predict OS for BLCA. The nomogram was evaluated using receiver operating characteristic (ROC) analysis and calibration plot in both the training and test sets. The area under curve [AUC] of the nomogram was calculated and compared with clinicopathological indicators using the full analysis set. Statistical analyses were conducted using the R software, where P-value <0.05 was considered significant.

**Results:**

The results indicated that age, race, sex, marital status, histology, tumor-node-metastasis (TNM) stages based on the AJCC 7th edition, and additional chemotherapy were independent prognostic factors for OS in patients with BLCA. Patients receiving chemotherapy tend to have better survival outcomes than those without. The proposed nomogram showed decent classification (AUCs >0.8) and prediction accuracy in both the training and test sets. Additionally, the AUC of the nomogram was observed to be better than that of conventional clinical indicators.

**Conclusions:**

The proposed nomogram incorporated independent prognostic factors including age, race, sex, marital status, histology, tumor-node-metastasis (TNM) stages, and additional chemotherapy. Patients with BLCA benefit from chemotherapy on overall survival. The nomogram-based prognostic model could predict overall survival for patients with BLCA with accurate stratification, which is superior to clinicopathological factors.

## Introduction

Bladder cancer (BLCA) accounts for the most common urinary malignancy with high mortality rate (1). Statistics showed that up to a quarter of BLCA cases are muscle-invasive or metastatic (2), while non-muscle-invasive BLCA has high progression and recurrence rates (3, 4). Surgery is indicated for non-metastatic BLCA, with transurethral resection of bladder tumor (TURBT) for the non-muscle-invasive and radical cystectomy (RC) for the muscle-invasive. For non-muscle-invasive BLCA with intermediate to high risk, intravesical chemotherapy is one of the first-line treatments (5, 6). For nonmetastatic muscle-invasive disease, neoadjuvant cisplatin-based chemotherapy followed by RC or chemoradiation combined with maximal TURBT are recommended (7); however, the overall 5-year survival rate of these patients remains less than 50% (8–10). Metastatic muscle-invasive disease can be treated with systemic chemotherapy and immunotherapy, which yields 5-year survival rates of 5% with distant metastasis and 36% with regional metastasis (7). In general, the overall survival of BLCA remains relatively low despite multiple treatment modalities. Therefore, it is important to develop prognostic model for overall survival of BLCA patients, as identifying patients with poor estimated survival outcomes may guide enhanced therapies for these subjects in an effort to improve prognosis (11).

In most clinical settings, prognostic estimates of patients with bladder cancer rely on the American Joint Commission on Cancer (AJCC) tumor-node-metastasis (TNM) staging system (12). While the AJCC staging has significance on evaluation of tumor burden, prognostic stratification, as well as on treatment, the system does not take demographic information into consideration (13). Further, additional treatment has shown impacts on survival chance for patients with BLCA, which should be considered in clinical prediction models for evaluating prognosis (14, 15). Researchers have injected tremendous enthusiasm into gene expression studies for prognostic models based on surgical samples of BLCA resection (16–18). However, batch effects on sequencing data *via* different platform are objective barriers for real-world validation, even with multiple statistical adjustment (19–21). Further, gene expression data in local surgical centers are not always accessible, especially in the remote area. Prediction models with clinical information available may offer a broader application in real world.

The surveillance, epidemiology, and end results (SEER) database incorporates data on diagnosis, treatment, and prognosis of cancer collected from 18 cancer registries which consists of 35% of US population. The database offers a platform for prognostic models in cancer patients with de-identified case lists. The aim of the present study was to establish a prognostic model for BLCA based on demographic information, AJCC staging, and additional treatment using the SEER data.

## Materials and Methods

### Patient Selection

Case lists were accessed from the SEER database using SEER*Stat version 8.3.6. Cases with bladder cancer diagnosed from 2010–2015 were included, while patient records with incomplete information on pre-specified variables were excluded. The pre-specified variables were as follows: age, race, sex, marital status, year of diagnosis, Tumor grade, Histology, TNM stages based on the AJCC 7th edition, radiation, chemotherapy, vital status, and survival month. Patients with age <18 years or survival time <30 days were excluded.

### Variable Coding and Statistical Analysis

Patient age was categorized into four classes, i.e., <60 yrs, 60–69 yrs, 70–79 yrs, and 80+ yrs. Marital status was coded as: married, unmarried, and SDW, which is short for single, divorce or separated, and widowed. There were four classes in tumor grade as well as in T stage (AJCC 7th edition), with the former being G1–G4 and the latter being T1/Ta/Tis, T2, T3 and T4. Variables including histology, N stage, M stage, radiation, and chemotherapy were coded as binary variables. Histology was classified into transitional cell papillomas/carcinomas, and Non-transitional; N stage was categorized into N0 and N1–3, while M stage (metastasis), radiation, and chemotherapy were coded into Yes or No.

All eligible cases were included in the full analysis set, which was then split into training set and test set with a 1:1 ratio. For each categorical variables, number and proportion of cases in each category were calculated in the three datasets. For continuous variable, median and interquartile interval were calculated in three datasets. Using the training set, univariate and multivariate Cox regression analyses were conducted to identify prognostic factors for overall survival in BLCA patients. Univariate Cox regression is a classical method for identifying prognostic factors using survival data with time and events, but there could be false-positive among the prognostic factors selected due to confounding effects (22), which can be corrected using multivariate Cox regression (23, 24). Kaplan–Meier curves were plotted to visualize the difference of survival rates as defined by categorical variables of interest. Contingency tables were analyzed between additional treatments and N stage/M stage to identify interaction using mosaic plots with independence chi-square test. Blue tiles in the mosaic plot represents more frequency than expected in the null model, while red tiles represent less frequency than expected. Treatment variables with significant interaction across different cancer stage were excluded, and other prognostic factors were selected for further analysis.

Subsequently, we formulated a nomogram with prognostic factors using the rms R package. Receiver operating characteristics (ROC) analysis was performed, and area under curves (AUCs) at 1-, 3-, and 5-year were calculated. AUCs >0.7 was considered acceptable classification. Calibration plot was performed to evaluate the prediction accuracy by comparing nomogram-predicted survival with actual survival in the training set. If the point estimates and error bar distributed close to the diagonal line where predicted survival equals to actual survival, then the nomogram was considered accurate. Likewise, ROC analysis and calibration were performed in the test set for validation. At last, the nomogram-based AUC were calculated and compared with clinicopathological indicators using the full analysis set. All statistical analyses were performed using the R software (www.r-project.org), and p values < 0.05 were considered statistically significant.

## Results

### Characteristics of Eligible Patients

**Figure 1:**
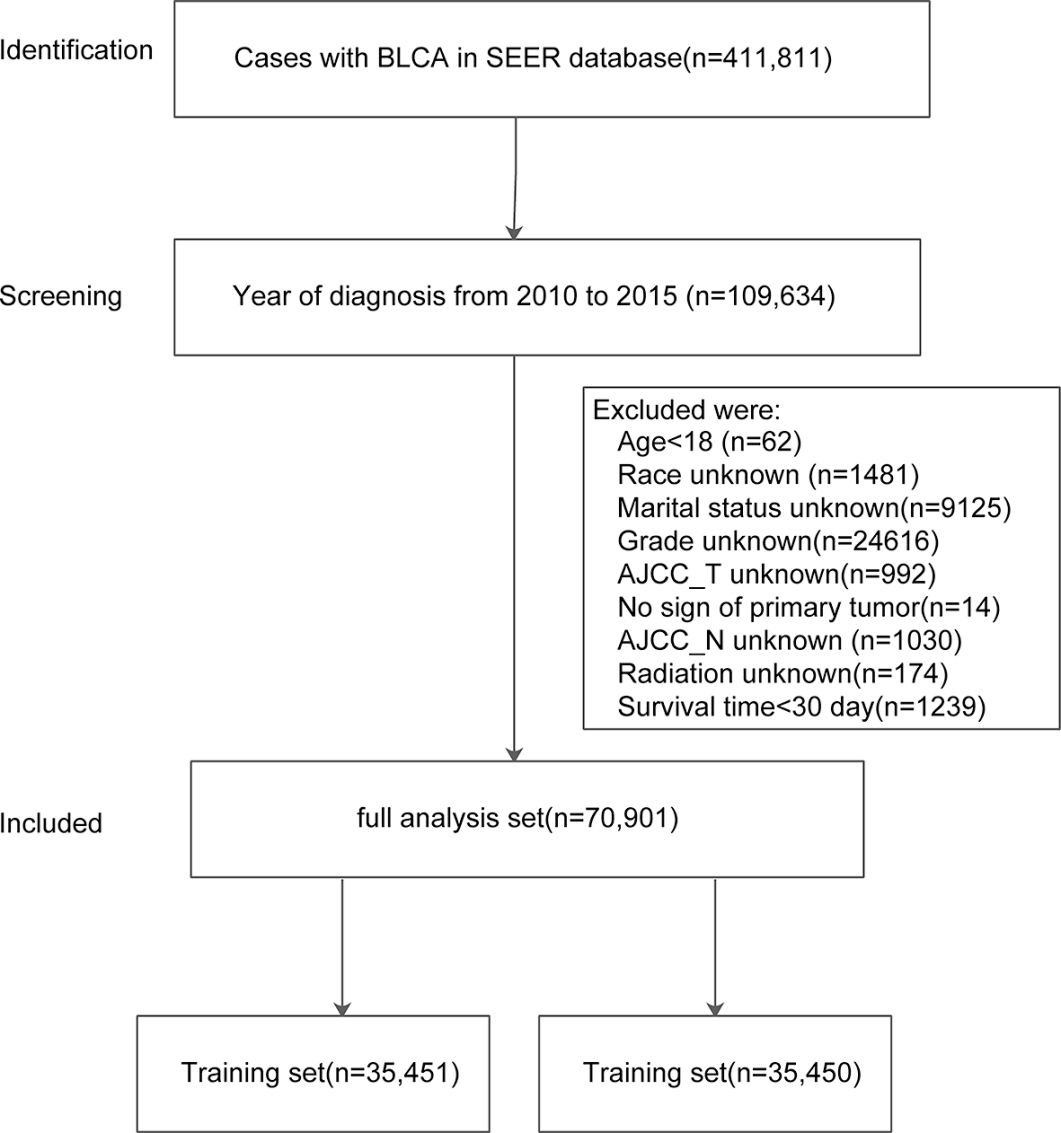
Flowchart of patient selection and dataset formulation. Of 411,811 BLCA cases in SEER, 109,634 cases diagnosed from 2010–2015 were screened with 70,901 included in the analyses.

Out of 411,811 cases, a total 109,634 cases were diagnosed with BLCA between 2010 and 2015 were identified in the SEER database. After excluding data according to aforementioned criteria, we retrieved patients records of 70,901 cases with BLCA (full analysis set). The full analysis set was then split into training set (n = 35,451) and test set (n = 35,450). The process of patient selection and dataset classification was presented in **Figure 1**. The characteristics of eligible BLCA cases were listed in **Table 1**. The proportions of cases among different categories were similar in three datasets. In the full analysis set, 15.66% of patients were at age <60 yrs, 26.17% were at 60–69 yrs, 30.42% at 70–79 yrs, and 27.75% at age >80 yrs. About 29% of cases received chemotherapy, while only 5.62% of cases received radiation. The median survival month was 30 months, with the interquartile interval being 16–34.07 months.

**Table 1:** Counts and proportions on the characteristics of eligible BLCA cases.

Variable	level	value	Train	Test
Age	<60	11,100 (15.66%)	5,616 (15.84%)	5,484 (15.47%)
	60–69	18,556 (26.17%)	9,246 (26.08%)	9,310 (26.26%)
	70–79	21,569 (30.42%)	10,838 (30.57%)	10,731 (30.27%)
	80+	19,676 (27.75%)	9,751 (27.51%)	9,925 (28%)
Race	Black	4,252 (6%)	2,187 (6.17%)	2,065 (5.83%)
	Other	3,222 (4.54%)	1,590 (4.49%)	1,632 (4.6%)
	White	63,427 (89.46%)	31,674 (89.35%)	31,753 (89.57%)
Sex	Female	16,597 (23.41%)	8,403 (23.7%)	8,194 (23.11%)
	Male	54,304 (76.59%)	27,048 (76.3%)	27,256 (76.89%)
Marital	Married	44,882 (63.3%)	22,306 (62.92%)	22,576 (63.68%)
	SDW	17,637 (24.88%)	8,922 (25.17%)	8,715 (24.58%)
	unmarried	8,382 (11.82%)	4,223 (11.91%)	4,159 (11.73%)
year of diagnosis	2010	11,028 (15.55%)	5,508 (15.54%)	5,520 (15.57%)
	2011	10,771 (15.19%)	5,305 (14.96%)	5,466 (15.42%)
	2012	11,205 (15.8%)	5,634 (15.89%)	5,571 (15.72%)
	2013	11,791 (16.63%)	5,877 (16.58%)	5,914 (16.68%)
	2014	12,724 (17.95%)	6,447 (18.19%)	6,277 (17.71%)
	2015	13,382 (18.87%)	6,680 (18.84%)	6,702 (18.91%)
Grade	1	9,334 (13.16%)	4,667 (13.16%)	4,667 (13.17%)
	2	17,802 (25.11%)	8,901 (25.11%)	8,901 (25.11%)
	3	12,271 (17.31%)	6,136 (17.31%)	6,135 (17.31%)
	4	31,494 (44.42%)	15,747 (44.42%)	15,747 (44.42%)
Histology	Non-transitional	2,671 (3.77%)	1,397 (3.94%)	1,274 (3.59%)
	transitional	68,230 (96.23%)	34,054 (96.06%)	34,176 (96.41%)
AJCC_T	T1/Ta/Tis	52,192 (73.61%)	26,050 (73.48%)	26,142 (73.74%)
	T2	12,397 (17.48%)	6,275 (17.7%)	6,122 (17.27%)
	T3	3,618 (5.1%)	1,810 (5.11%)	1,808 (5.1%)
	T4	2,694 (3.8%)	1,316 (3.71%)	1,378 (3.89%)
AJCC_N	N0	67,252 (94.85%)	33,708 (95.08%)	33,544 (94.62%)
	N1–3	3,649 (5.15%)	1,743 (4.92%)	1,906 (5.38%)
AJCC_M	M0	68,730 (96.94%)	34,323 (96.82%)	34,407 (97.06%)
	M1	2,171 (3.06%)	1,128 (3.18%)	1,043 (2.94%)
Radiation	No	66,914 (94.38%)	33,437 (94.32%)	33,477 (94.43%)
	Yes	3,987 (5.62%)	2,014 (5.68%)	1,973 (5.57%)
Chemotherapy	No	50,341 (71%)	25,171 (71%)	25,170 (71%)
	Yes	20,560 (29%)	10,280 (29%)	10,280 (29%)
Vital_status	Alive	46,518 (65.61%)	23,222 (65.5%)	23,296 (65.72%)
	Dead	24,383 (34.39%)	12,229 (34.5%)	12,154 (34.28%)
survival_month		30 [16,34.07]	30 [16,34.01]	30 [16,34.12]

### Selection of Prognostic Factors

**Figure 2:**
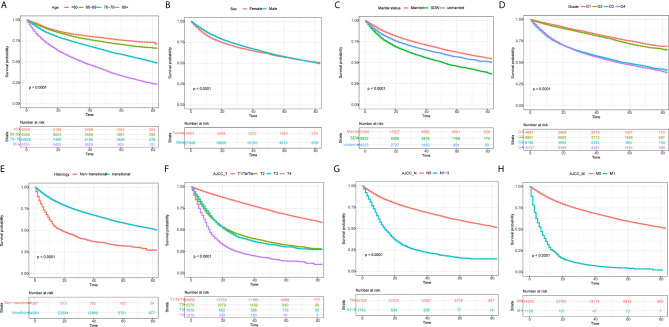
Kaplan–Meier curves for overall survival in BLCA patients on different stratification. **(A)** age; **(B)** Sex; **(C)** marital status; **(D)** grade; **(E)** histology; **(F)** AJCC_T stage; **(G)** AJCC_N stage; **(H)** AJCC_M stage. Kaplan–Meier curves with different colors represent survival status on given subgroup, while risk table below the curves records the number of cases at specific follow-up time. All P-values were <0.0001.

Apart from year of diagnosis which is not applicable for prediction, we exploited all variables into univariate and multivariate Cox regression models using the training set. The results showed that age, race, sex, marital status, histology, TNM stages based on the AJCC 7th edition, radiation, and chemotherapy were prognostic factors for overall survival in patients with BLCA (**Table 2**). For instance, higher age (HRs >1, P <0.001), SDW or unmarried status (HR = 1.349, 95% CI: 1.294–1.407, P <0.001), higher TNM stages based on AJCC 7th edition were associated with worse survival rates. By contrast, male patients, or patients with transitional cell papillomas/carcinomas were associated with favorable survival chance. Compared to G1 patients, G2 patients do not exhibit a distinct survival (P = 0.1023), while G3 and G4 patients reported worse survival (HRs >1, P <0.001). Kaplan–Meier curves on variables of interests were presented in **Figure 2**, where log-rank tests showed similar results to Cox regression.

**Table 2:** Results of univariate and multivariate Cox regression using the training set.

Id		Train_unicox				Train_multicox			
		HR	HR.95L	HR.95H	p-value	HR	HR.95L	HR.95H	p-value
Age	<60								
	60–69	1.23	1.14	1.32	<0.0001	1.31	1.22	1.41	<0.0001
	70–79	1.83	1.71	1.96	<0.0001	1.96	1.83	2.10	<0.0001
	80+	3.71	3.48	3.96	<0.0001	3.83	3.58	4.10	<0.0001
Race	Black								
	White	0.66	0.59	0.74	<0.0001	1.09	1.04	1.13	0.0002
	other	0.74	0.69	0.79	<0.0001	0.69	0.62	0.77	<0.0001
Sex	Female								
	Male	0.91	0.88	0.95	<0.0001	0.80	0.75	0.86	<0.0001
Marital_status	Married								
	SDW	1.72	1.65	1.79	<0.0001	1.35	1.29	1.41	<0.0001
	Unmarried	1.25	1.19	1.33	<0.0001	1.39	1.31	1.47	<0.0001
Grade	1								
	2	1.13	1.04	1.22	0.0031	1.07	0.99	1.16	0.1023
	3	2.82	2.62	3.04	<0.0001	1.65	1.53	1.78	<0.0001
	4	2.91	2.72	3.12	<0.0001	1.66	1.55	1.79	<0.0001
Histology	Non-Transitional								
	transitional	0.38	0.36	0.41	<0.0001	0.63	0.59	0.68	<0.0001
AJCC_T	T1/Ta/Tis								
	T2	3.56	3.41	3.70	<0.0001	2.53	2.41	2.65	<0.0001
	T3	3.68	3.45	3.92	<0.0001	2.77	2.59	2.98	<0.0001
	T4	6.47	6.06	6.92	<0.0001	3.95	3.66	4.26	<0.0001
AJCC_N	N0								
	N1–3	3.90	3.68	4.13	<0.0001	1.54	1.44	1.65	<0.0001
AJCC_M	M0								
	M1	7.76	7.27	8.29	<0.0001	3.52	3.27	3.79	<0.0001
Radiation	No								
	Yes	3.25	3.08	3.44	<0.0001	1.26	1.18	1.34	<0.0001
Chemotherapy	No								
	Yes	1.17	1.12	1.21	<0.0001	0.75	0.72	0.78	<0.0001

**Figure 3:**
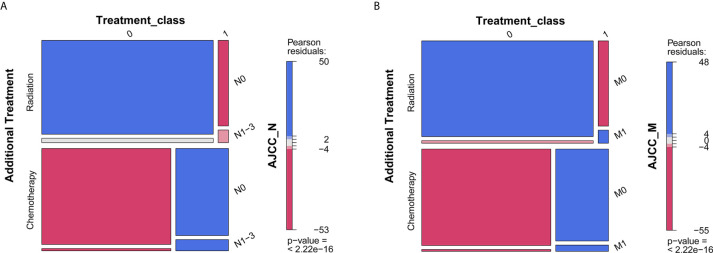
Mosaic plot for describing interaction among additional treatment, treatment class, and AJCC stages. **(A)** AJCC_N stage; **(B)** AJCC_M stage. P < 0.05 indicates interaction between variables. The blue tiles represent more frequency than expected in the null model, while red tiles represent less frequency than expected.

What should be noted is that radiation seems to be associated with worse survival, while chemotherapy was associated with favorable survival (**Table 2**). Given that patients with positive lymph node or metastasis tend to receive additional treatment (radiation or chemotherapy), we conducted contingency table analysis to identify potential interactions of treatment across different cancer stages (AJCC_N or AJCC_M). As shown in **Figure 3A**, there was systematic association among additional treatment, treatment class and AJCC_N stage (P <2.2 ∗ 10^−16^). More cases with negative lymph node than expected did not receive radiation, while patients tend to receive chemotherapy (as compared to radiation) whether there was positive lymph node or not. Likewise, **Figure 3B** showed that interaction existed among therapy item, therapy class and AJCC_M stage (P <2.2 ∗ 10^−16^). Patients without metastasis tend to receive no radiation, while those with metastasis tend to receive radiation; whereas, patients tend to receive chemotherapy (as compared to radiation) whether there was metastasis or not. Given that only 5.62% of cases received radiation and significant interaction existed between radiation and status on lymph node and metastasis, whether the patient received radiation or not could not serve as valid predictor for overall survival. As such, we selected the following prognostic factors for further analyses: age, race, sex, marital status, histology, TNM stages based on the AJCC 7th edition, and chemotherapy.

### Development and Validation of a Prognostic Nomogram

**Figure 4:**
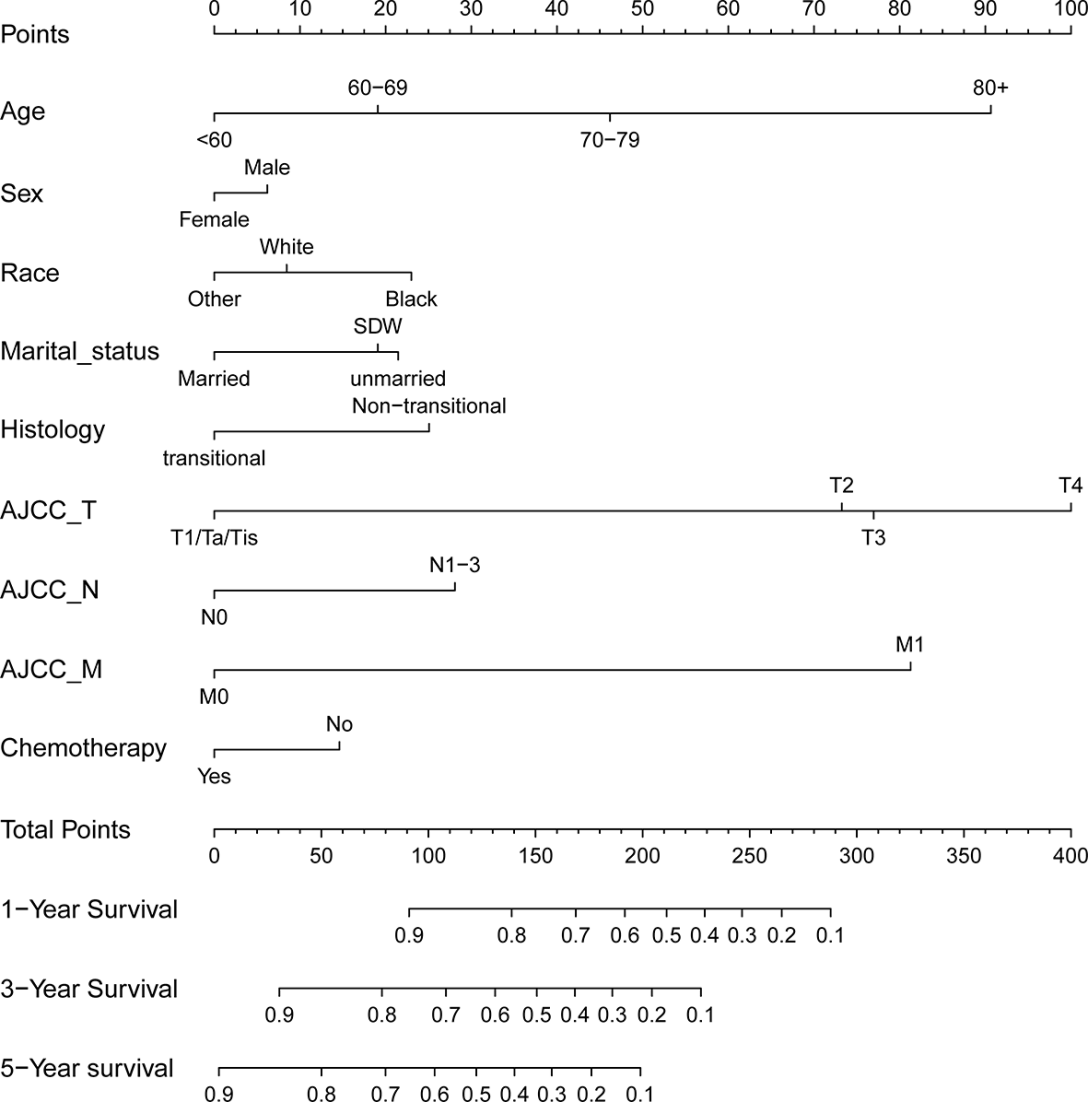
Nomogram for predicting 1-, 3-, and 5-year overall survival rates for BLCA patients. For individual patient, a score was assigned based on each factor in the nomogram, and total points could be calculated and corresponded vertically to survival probability using the nomogram.

**Figure 5:**
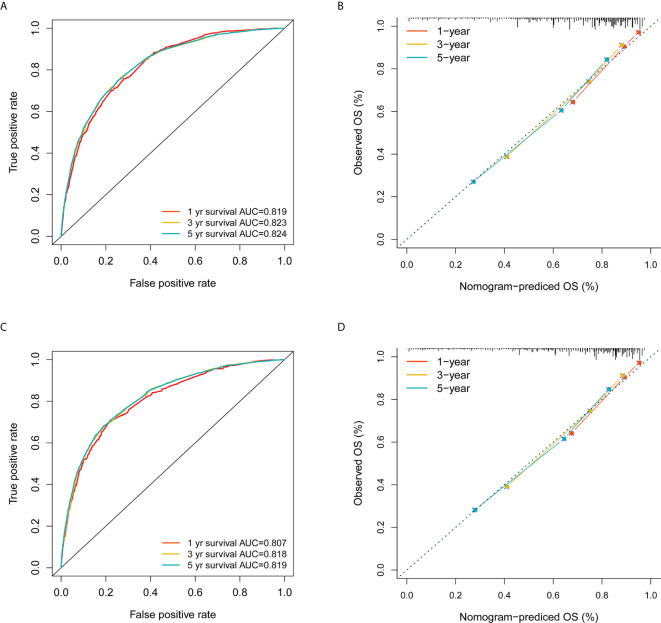
Validation of the nomogram in the training and test sets. **(A)** ROC analysis using the training set; **(B)** Calibration plot using the training set; **(C)** ROC analysis using the test set; **(D)** Calibration plot using the test set. The red, yellow, and blue curves represent 1-, 3- and 5-year survival classification in **(A, C)**; The three colors also represent 1-, 3- and 5-year survival estimation based on actual observation in **(B, D)**, while the gray dashed line represent ideal calibration where the observed survival probability equals to the nomogram predictions.

**Figure 6:**
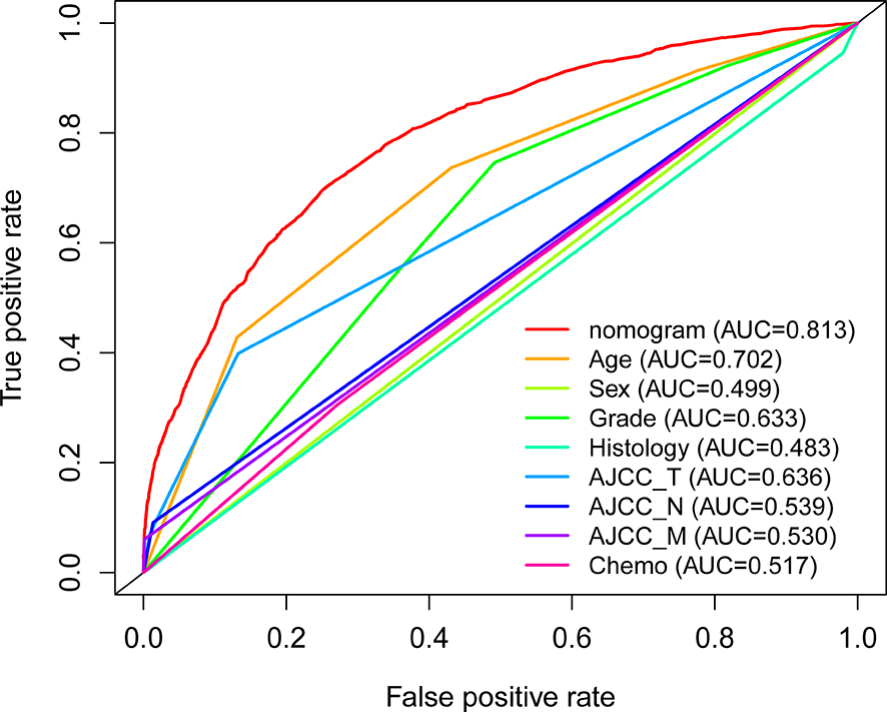
ROC analysis for multiple indicators using the full analysis set.

With the selected prognostic factor, we developed a prognostic nomogram using the training set. The nomogram was presented in **Figure 4**, where individualized survival chance at 1-, 3-, and 5-year could be predicted using accessible clinical information. Values for each variable correspond to nomogram points, and we can calculate total points by adding them up. Subsequently, the value of total points corresponds vertically to survival chances at multiple timepoints. The ROC curve analysis of the nomogram in the training set showed acceptable to excellent accuracy in classification with 1-year AUC of 0.819, 3-year AUC of 0.823, and 5-year AUC of 0.824 (**Figure 5A**). Additionally, ROC analysis in the test set validated the classification performance with 1-year AUC of 0.807, 3-year AUC of 0.818, and 5-year AUC of 0.819 (**Figure 5C**). Moreover, calibration plot revealed favorable prediction accuracy of the nomogram at multiple timepoints in both the training set (**Figure 5B**) and test set (**Figure 5D**). Besides, the AUC of the nomogram (0.813) was observed to be larger than that of age (0.702), sex (0.499), grade (0.633), histology (0.483), TNM stages (0.636), or chemotherapy (0.539) (**Figure 6**).

## Discussion

Our data indicated that age, race, sex, marital status, histology, TNM stages based on the AJCC 7th edition, and additional chemotherapy were independent prognostic factors for overall survival in patients with BLCA. With the independent prognostic factors, we established a nomogram with decent classification and prediction accuracy in both the training and test sets. Further, the AUC of the nomogram was observed to be better than that of clinicopathological factors. The proposed nomogram model could serve as a guidance for prognostic estimates for patients with BLCA in clinical practice, thereby facilitating shared decision-making among different stakeholders on BLCA.

According to the results, BLCA in the elderly seems to yield worse survival as opposed to younger patients (age <60 yrs). Consistent with a previous report, African Americans (Race: Black) was associated with worse survival rates as compared to white people, native Americans, and Asian Americans (25). In comparison to SDW and unmarried patients, married patients have better prognosis. The protective effects of married marital status have been reported extensively (26–28). Most of BLCA cases were male; however, female patients tend to have worse survival. The impact of gender on survival chance for BLCA was consistent with a previous study (29). The most common histology is transitional cell papillomas/carcinomas, which is correlated with favorable survival compared to other types. Additional chemotherapy was associated with favorable overall survival in BLCA cases after clinicopathological information was corrected. Significantly favorable survival associated with platinum-based neoadjuvant chemotherapy established in the 1970s for advanced bladder cancer was observed in previous studies (30–32). Additional chemotherapy with Bacillus Calmette–Guérin (BCG) to surgery has also shown superior survival outcomes to surgery alone for non-muscle-invasive bladder cancer (NMIBC) (33). These results align with our findings on the impact of additional chemotherapy. According to the multivariate Cox regression, radiation appears to have detrimental effects on overall survival, which could be attributed to its interaction with AJCC lymph node and metastasis stages. Likewise, the previous nomogram model did not detect the independent prognostic value of radiation therapy (11). The effects of additional radiation therapy for BLCA require further investigation.

The present study established a nomogram to visualize the individualized survival chances of BLCA patients with the selected prognostic factors using the training set. The nomogram has shown good classification as well as prediction accuracy in both the training set and test set. Further, the AUCs (>0.80) were superior to previous prognostic model (11, 27, 34) for BLCA patients. The optimized classification accuracy could be partly due to the increased sample size in the present study. Besides, the aforementioned studies have confined subjects to those undergoing radical cystectomy, which limits the generalizability of the models as patients could receive different surgical procedure. In contrast, the present study included all BLCA patients with complete prespecified information, resulting in a broader applicability of the present model. While our model applies to broader subjects, criticism may follow on the heterogeneity of patients receiving different treatment; however, the optimized classification accuracy has proved homogeneity to some extent. To our best knowledge, the present study is the first report of accurate nomogram model tailored for BLCA without limits on specific surgical procedures.

Recently, mounting studies using RNA sequencing data have been proposed to investigate the prognosis of patients with BLCA (35–37). These studies adopted transcriptomic data based on mRNA/lncRNA expressions for prognostic models; however, sequencing data are not always accessible, usually expensive, and subject to batch effects on different sequencing modalities. Gene signatures on BLCA were reported extensively related to different gene sets on immune response (18), epithelial‐mesenchymal transition (EMT) signaling (36), and glycolysis (38). These signatures were developed and validated, with limited discriminative accuracy (AUCs from 0.60 to 0.77). Therefore, the applicability and accessibility appear to be insufficient for prediction models using gene expression profiles. The goal of prediction models is to bridge knowledge gaps across different stakeholders with simple and accessible information (39, 40). In this regard, our model exploited demographic, pathologic, and clinical data to build a nomogram model for overall BLCA patients, which could be an accessible tool for prognostic evaluation in clinical practice.

Notably, our study has a few limitations. First, preoperative laboratory results, surgical margin information, and comorbidity were not accessible in the SEER database, which may limit the predictive performance of the present model. Therefore, the proposed prognostic model can be considered only as preliminary for further analyses where the contribution of patient comorbidity can be modeled and correctly applied. Second, we conducted complete case analysis; as such, selection bias may have been introduced despite the small proportion. However, the population-based design with a considerable sample size has ensured the robustness of our results to some degree. Prospective clinical studies with rigorous design are still needed for external validation.

Further research should investigate novel imaging application tools for the prediction of BLCA survival outcomes, as the role of multiparametric MRI (mpMRI) within nomograms has been demonstrated in prostate cancer (41, 42). Besides, the newly released Vesical Imaging-Reporting and Data System (VI-RADS) based on mpMRI data has shown promises for accurate preoperative BLCA staging (43–45), which could be exploited for the estimation of cancer-specific and overall survival. These imaging-based assessments could be incorporated in prognostic nomograms in the future.

## Conclusion

Age, race, sex, marital status, histology, tumor-node-metastasis (TNM) stages based on the AJCC 7th edition, and additional chemotherapy were independent prognostic factors for OS in patients with BLCA. Additional chemotherapy (as compared to radiation) seems to be independent of whether there was positive lymph node/metastasis or not; those receiving chemotherapy have better survival outcomes. The nomogram based on these prognostic factors was observed to be more accurate on overall survival estimation than clinicopathological factors. However, prospective studies are warranted for external validation.

## Data Availability Statement

Publicly available datasets were analyzed in this study. The data can be accessed on SEER database (https://seer.cancer.gov/).

## Author Contributions

WW, JL, and LL contributed to data processing, interpretation of results, and drafting. LL supervised the study and approved the draft. All authors contributed to the article and approved the submitted version.

## Funding

The present study was funded by Research on the key technology of constructing basic database related to war trauma and environment (19-163-12-ZT-006-004-02) and Research on the technology and construction of regional rehabilitation medical system for navy officers and ratings (20BJZ08).

## Conflict of Interest

The authors declare that the research was conducted in the absence of any commercial or financial relationships that could be construed as a potential conflict of interest.
